# Point-Counterpoint: The “obsoletogram”—are annual hospital antibiograms still relevant?

**DOI:** 10.1128/jcm.00476-26

**Published:** 2026-05-22

**Authors:** Zoe Freeman Weiss, Sankha S. Basu

**Affiliations:** 1 Division of Geographic Medicine and Infectious Diseases, Tufts Medical Center 1867, Boston, Massachusetts, USA; 2 Department of Pathology and Laboratory Medicine, Tufts Medical Center 1867, Boston, Massachusetts, USA; 3 Department of Pathology, Brigham and Women’s Hospital 1861, Boston, Massachusetts, USA; Vanderbilt University Medical Center, Nashville, Tennessee, USA

## Abstract

Annual hospital antibiograms, cumulative reports of antimicrobial susceptibility data from clinical isolates, are widely used to guide empiric antibiotic therapy, monitor resistance trends, and support antimicrobial stewardship. Most hospital antibiograms are compiled annually from clinical bacterial isolates, using the first isolate per patient and species. Although antibiograms have long proven their value, some now question whether traditional versions still meet modern clinical needs, given the need for personalized medical decision-making and emerging new diagnostic technologies that can provide faster, actionable results. In the following point-counterpoint article, the limitations of traditional antibiograms and the view that they may no longer be adequate for contemporary surveillance or decision-making are contrasted with the reasons antibiograms may remain essential and how they can evolve to stay relevant in modern healthcare.

## THE ROLE AND FUTURE OF HOSPITAL ANTIBIOGRAMS

Annual hospital antibiograms, cumulative reports of antimicrobial susceptibility data from clinical isolates, are widely used to guide empiric antibiotic therapy, monitor resistance trends, and support antimicrobial stewardship. In 2006, the Clinical and Laboratory Standards Institute (CLSI) M39 guideline on the creation of an annual antibiogram (1) was introduced. This document provides guidance on how to collect data, analyze, generate, and present cumulative antibiograms, and undergoes review and revision every 5 years (2).

Most hospital antibiograms are compiled annually from clinical bacterial isolates, using the first isolate per patient and species. Following CLSI M39 guidelines (1), data are aggregated to show the percentage of susceptible isolates for each antimicrobial agent, typically including only organisms with at least 30 isolates to maintain statistical validity. Institutions may stratify their reports by specimen type, hospital unit, or patient population. Basic antibiograms give local antimicrobial susceptibility data in a familiar and straightforward format that clinicians, pharmacists, and infection prevention staff can easily interpret (3). Most regulatory and public health agencies require clinical laboratories to compile and submit cumulative antimicrobial susceptibility data, often in the form of institutional antibiograms, for surveillance and quality assurance purposes. For example, the Centers for Disease Control and Prevention’s National Healthcare Safety Network Antibiotic Resistance module enables facilities to report antimicrobial susceptibility results, which is a requirement for those hospitals that participate in the Medicare Promoting Interoperability Program (https://www.cdc.gov/nhsn/cms/cms-faq-aur.html).

Although antibiograms have long proven their value, some now question whether traditional versions still meet modern clinical needs, given the need for personalized medical decision-making and emerging new diagnostic technologies that can provide faster actionable results. In the following point-counterpoint article, the limitations of traditional antibiograms and the view that they may no longer be adequate for contemporary surveillance or decision-making are contrasted with the reasons antibiograms may remain essential and how they can evolve to stay relevant in modern healthcare.

**Romney M. Humphries, Point-Counterpoint Editor,** ***Journal of Clinical Microbiology***

## POINT

### Hospital antibiograms will soon be obsolete

Each year, hospital groups made up of microbiologists, pharmacists, and antimicrobial stewardship members work together to update and validate their cumulative hospital antibiograms. While these antibiograms have grown in importance as public health tools to monitor and better understand local resistance trends, their primary purpose is to guide initial antimicrobial therapy. Still, despite their widespread utilization, they are challenging and resource-intensive to generate, can lead to biased or inaccurate resistance rates, require nuanced clinical interpretation, and there is little evidence that they benefit patients or improve clinical outcomes (4–6). Moreover, newer diagnostic platforms provide earlier susceptibility results, shortening the empiric therapy “window,” while emerging AI-based tools may provide better guidance than traditional antibiograms, raising the question of whether the considerable effort spent to maintain cumulative hospital antibiograms is still warranted.

### Antibiograms are challenging and resource- intensive to generate

Although the CLSI M39 provides guidelines for generating and reporting hospital antibiograms (1), the process is often labor-intensive due to non-uniform data structures, manual data cleaning, and the difficulty of combining results from different antimicrobial susceptibility testing (AST) platforms and modalities (e.g., automated vs manual). One 2015 study demonstrated that fewer than 1 in 10 community hospitals demonstrated full compliance with CLSI guidelines (4). Using automated approaches to generate antibiograms can streamline the process but requires additional validation (2, 4). Compounding these challenges is the impact of clinical breakpoint changes, which can shift susceptibility categorizations. If historical results are not reanalyzed using a single, consistent breakpoint set, aggregated antibiogram data may be inaccurate. Accurate longitudinal analysis often requires manual reclassification of the entire data set using updated breakpoints, which is labor-intensive and not routinely performed. Also, although infrequent, updates to the electronic medical record or laboratory information system can make it challenging to pool patient-level data, such as determining the first isolate per patient per year.

Annual updates, as typically performed, are too slow to guide real-time clinical decisions or detect rapid shifts in resistance. Infection prevention programs must rely on continuous isolate tracking and unit-specific multi-drug resistant organism surveillance to detect and respond promptly to emerging threats. The CLSI M39 guideline’s limitation to the first isolate per patient per species also causes these reports to obscure unit-level outbreaks and short-term resistance trends (1). While M39 does recommend more frequent preparation of antibiograms, most hospitals generate these annually (2, 7) due to the resources needed to generate them. Because annual antibiograms are static summaries, they have limited utility for detecting rapid shifts in resistance patterns or informing individual patient-level decisions.

### Antibiograms may provide inaccurate resistance rates

The data and processes used to generate antibiograms often lead to biased and inaccurate resistance rates due to multiple factors. The first involves an unintended consequence of antimicrobial stewardship efforts where “cascading” susceptibility testing or reporting is utilized. With cascading results, certain antimicrobial agents may not be routinely tested or reported in the record unless the more narrow or preferred drugs demonstrate resistance. As a result, resistance rates may appear higher due to selective testing or reporting. Conversely, if resistant results are withheld (e.g., due to an instrument or product limitation), the aggregated isolates may artificially appear more susceptible than they really are.

Another factor relates to the generalizability of antibiogram results. Most hospitals typically prepare a single antibiogram where susceptibility data are aggregated across patient populations, units, and time (usually annually), failing to reflect specific resistance patterns relevant to individual units, syndromes, or patients. These strategies do not capture source-specific or strain-level differences that could be used to personalize decision-making. They also do not account for critical patient-specific factors such as prior colonization, social history (e.g., recent exposure to a long-term care environment), recent antibiotic use, or comorbid conditions. Antibiograms also do not capture the temporal relationship between specimen collection and hospital admission. Without that context, the data cannot support accurate classification of infections as community onset or healthcare associated (2).

Pooling isolates across specimen types introduces additional limitations. An isolate recovered from urine may not be clinically comparable to the same species isolated from the lung, where antibiotic penetration differs and local treatment practices apply different selective pressures. Separately, the epidemiology of organisms differs by specimen type. Highly resistant colonizing organisms, such as *Pseudomonas aeruginosa*, *Acinetobacter baumannii*, and *Stenotrophomonas maltophilia*, are frequently recovered from endotracheal aspirates and may overrepresent resistance when included alongside isolates truly causing invasive disease. Interestingly, it is also not uncommon for isolates to appear more susceptible in invasive disease than in colonization, raising concerns about trends toward overuse of empiric broad-spectrum therapies (8). Unit-specific antibiograms also have limitations: different patient populations within an intensive care unit (ICU; e.g., medical vs surgical, short vs prolonged stay) can exhibit varying resistance patterns, limiting the predictive value of aggregate data. The CLSI-recommended practice of including only the first isolate per patient may underestimate rising resistance in the ICU setting. Additionally, unit-specific or source-specific (e.g., respiratory isolates only) antibiograms are often sparse due to the limited number of organisms isolated from smaller unit populations.

### Antibiograms are ineffective at guiding treatment

It may seem in principle that using local resistance rates would help guide effective therapy, but empirical data suggest that the clinical utility may be limited. For example, Hasegawa et al. (9) assessed the diagnostic accuracy of hospital antibiograms for *Enterobacterales* and found them to be relatively poor at predicting AMR resistance, with receiver operating curve values ranging between 0.576 and 0.715 across different antibiotic classes. Similar findings were also seen for gram-positive organisms, with limited improvement when considering seasonal or geographical variation (10).

Clinical providers use antibiograms to guide empiric therapy, often using the “90% rule” (6), which suggests empiric antibiotic use only if ≥90% of isolates within a particular antimicrobial/organism combination are susceptible on the antibiogram. While this heuristic is intended to be applied alongside clinical judgment, it can oversimplify decision-making by de-emphasizing patient-specific factors such as immune status, prior antibiotic exposure, comorbid conditions, pharmacokinetic and pharmacodynamic considerations, and infection site. For example, a provider caring for a diabetic patient with recurrent urinary tract infections (UTIs) and prior *Escherichia coli* infections treated with quinolones might see 91% ciprofloxacin susceptibility on the antibiogram, yet their individual risk factors make resistance more likely, making ciprofloxacin a poor empiric choice.

Antibiograms are also frequently used to inform institution-specific treatment guidelines and formulary decisions. Recommendations derived from aggregated antibiogram data may not align with the patient populations or syndromes targeted by specific guidelines, as hospital-wide summaries can obscure differences between inpatient and outpatient settings or units with varying antimicrobial pressure, and when used in isolation may drive formulary or guideline decisions that fail to reflect patient heterogeneity, resistance risk factors, or infection site considerations. As a result, policies derived primarily from aggregate susceptibility data may unintentionally promote overtreatment in some populations and undertreatment in others (11).

The pooled, retrospective nature of cumulative antibiograms obscures nuances relevant to high-risk patients and offers limited guidance for tailoring empirical therapy in complex scenarios. Dynamic tools such as Bayesian forecasting and machine learning could be better suited for guiding empirical therapy in these settings.

### Technological advancements make antibiograms less relevant

Perhaps most importantly, recent and ongoing developments in AST platforms and bioinformatic tools make the use of antibiograms less relevant today. After all, the true benefit of the antibiogram in guiding empiric antimicrobial management lies in the window between organism identification and the availability of antibiotic susceptibility testing. During this window, knowledge of prevailing local resistance could be useful in guiding empiric therapy. This window will shorten as rapid phenotypic platforms are becoming commercially available. While conventional AST used to take 48–54 hours, rapid platforms can provide phenotypic results in 4.5–8 hours (12). Beyond this, newer nucleic acid-based molecular testing provides information on antimicrobial resistance genes alongside organism identification, often within an hour or two of a positive blood culture (e.g., *mecA* gene detection in *Staphylococcus aureus* or a CTX-M in an *E. coli*). Finally, predicting resistance and susceptibility through genotypic approaches has proven to be more powerful and accurate than initially believed and will further improve upon current approaches (13). As these technologies continue to evolve, the critical window during which the antibiogram is useful will close further.

Finally, one of the most compelling developments which may obviate the need for antibiograms is the emergence of artificial intelligence (AI) and machine learning tools that predict drug susceptibility from a patient’s electronic health record, a development especially relevant to empiric treatment for UTIs (14). For example, Kanjilal et al. demonstrated that a machine learning algorithm could reduce the use of second-line antibiotics for UTI by 67% (15), while Lee et al. demonstrated a potential reduction in the utilization of an ineffective antibiotic by 20% (16). Additional studies using similar approaches demonstrated that such algorithms could specifically predict the susceptibility toward several drugs commonly used to treat UTIs (17–21) or to predict the emergence of multi-drug resistance UTIs simply by analyzing common demographic variables (21, 22).

As diagnostic technology and predictive analytics advance, static and retrospective susceptibility summaries add little value beyond regulatory compliance. Antibiograms are slow, coarse, and disconnected from the clinical realities of individualized medicine. Maintaining them consumes time and resources that could instead support real-time patient-level predictive tools and investment in rapid point-of-care technologies. Within a few years, the antibiogram will be a relic of the past, useful for historical surveillance but irrelevant for modern empiric therapy or infection control.

**Sankha S. Basu**

## COUNTERPOINT

### Hospital antibiograms are here to stay

#### Still relevant for empiric therapy

Every hospital or healthcare system depends on antibiogram data more than it realizes. Although novel diagnostics continue to advance toward point-of-care use, there will always be scenarios in which antibiotics must be initiated before susceptibility information is available or in the absence of any data at all. Antibiograms provide guidance for treatment of infections for which cultures are never obtained, such as in outpatients presenting with uncomplicated UTI or community-acquired pneumonia. Antibiograms help guide empiric protocols for these scenarios as well as high-risk infections, such as febrile neutropenia, diabetic foot infections, sacral osteomyelitis, or healthcare-associated pneumonia. As an example, 50%–80% of patients with febrile neutropenia are culture negative (23–25). Local *Pseudomonas* susceptibility rates inform empiric gram-negative coverage in this population. Diabetic foot infections and sacral wounds are often polymicrobial, and the growth of an organism may not represent all pathogens present (or may reflect skin flora rather than pathogens). Cultures may be negative if patients have been on prolonged therapy. Local resistance data help ensure these empiric choices are informed by the most likely susceptibility patterns (3, 26). For hospitals that use mass spectrometry to identify organisms from any source, organism identification is typically reported at least a day earlier than traditional AST results, leaving a consistent gap during which antibiogram data can guide empirical therapy (27).

Antibiograms serve as a core reference and teaching tool for clinicians to understand local susceptibility patterns and make rational, empiric choices. They are essential in supporting antimicrobial stewardship programs, providing a shared data source for communicating with clinicians about local susceptibility trends (informing empiric choices) and for evaluating the impact of stewardship actions on prescribing behavior and susceptibility trends (28).

While antibiograms remain central to empiric therapy, widely used and emerging diagnostic technologies aim to narrow the window to definitive organism-specific treatment (e.g., multiplexed rapid blood culture identification panels with resistance genes) (29). However, these platforms do not yet capture the full range of phenotypic resistance, particularly in gram-negative organisms (30). Rapid AST platforms can provide results in as little as 6 hours from positive blood cultures. However, empiric antimicrobial therapy is still required before these results are available (and blood cultures incubate for up to 5 days!). Given this delay and the limited scope of organisms and resistance genes included in panels, empiric antibiotic selection will continue to depend on cumulative antibiogram data to guide initial treatment decisions.

The use of genotypic data, though not comprehensive enough to replace AST, complements traditional antibiogram functions. The CLSI M39 guideline recommends adding genotypic resistance markers when they have a strong predictive value for resistance (e.g., *mecA* conferring methicillin resistance in *Staphylococcus* spp.) because this improves the interpretation of local susceptibility patterns (1). When genotypic results do not fully align with phenotype, cumulative antibiogram data still guide clinical recommendations. At my institution, for example, blood culture identification results showing *E. coli* without resistance genes trigger a comment recommending ceftriaxone for community-onset infections and cefepime for nosocomial or critically ill patients (pending final susceptibilities), which reflects local nosocomial rates of non-CTX-M-mediated ceftriaxone resistance.

#### Public health and quality tracking

Aggregated antibiogram data enable benchmarking across facilities, allowing comparison of resistance rates and identification of outliers. For example, a hospital with certain species demonstrating substantially lower percent susceptible to fluoroquinolones as compared to peer institutions could prompt targeted investigation, audit of prescribing patterns, or additional infection control measures. Hospitals routinely submit their isolate-level data to the National Healthcare Safety Network as well as cumulative antibiograms to local health departments, supporting large-scale assessments of susceptibility trends and policy development (7). The value of aggregated data has limits because institutions differ in the agents they test, the quality of submitted data, the breakpoints used, and the accuracy of species identification. While antibiograms are not explicitly used to track resistance, as their inclusion rules are designed for empiric therapy guidance rather than population-level monitoring, aggregated antibiograms offer a practical link between local stewardship activities and broader surveillance efforts (31). Failure to produce reliable antibiogram data or to follow standardized reporting practices weakens resistance tracking at local, national, and global levels.

Of note, CLSI M39 was developed primarily for cumulative antibiograms used to guide empiric therapy and recommends standardized isolate selection, typically the first isolate per patient per analysis period, whereas susceptibility data used for resistance surveillance address a different epidemiologic question and may require alternative inclusion strategies such as repeat isolates or organism-specific approaches. M39 permits alternative methods for surveillance analyses, provided the approach is defined in advance and applied consistently over time. Thus, antibiograms intended to guide empiric therapy and analyses designed to monitor resistance trends represent conceptually and operationally distinct uses of the same underlying data (1).

#### Supports formulary decisions

Institutional antimicrobial guidelines rely on local resistance data to determine which agents to include, restrict, or remove. At our institution, cumulative susceptibility data showed no added benefit of meropenem-vaborbactam over existing options for *Enterobacterales*, such as ceftazidime-avibactam, so it was not added to the formulary. Similarly, a recent cluster of ICU-associated *Acinetobacter* isolates accounted for nearly all carbapenem-resistant *Acinetobacter* isolates recovered at our institution over the preceding year. Notably, all isolates in this cluster were also resistant to sulbactam-durlobactam. Given our local epidemiology, this agent was not adopted for our preferred formulary despite its designation as a first-line agent and global data demonstrating that >96% of isolates are susceptible (32). Broader agents such as these are usually presented in enhanced or stewardship-focused antibiograms since they are not part of routine primary testing and are not necessarily included in standard institutional antibiograms. These data-driven decisions keep the formulary aligned with current hospital resistance patterns.

#### Modernization over replacement

Creating a basic antibiogram is a straightforward process that does not require any additional isolate testing beyond what is already done for clinical care. Critics argue that antibiograms are too static, lack patient-specific detail, and will be outpaced by more dynamic technologies and advanced decision support tools. However, many of these concerns reflect the limitations of implementation rather than a failure of the underlying concept.

Hospitals are increasingly developing tailored antibiograms focused on specific units, pathogens, or clinical syndromes. Despite their availability, antibiograms may be underutilized by frontline clinicians due to limited awareness, lack of training, or difficulty accessing reports (33). Integrating antibiogram data into clinical pathways and decision support systems (CDSS) can help address this gap (3, 34). Electronic antibiograms can provide data stratification by infection source, acquisition type, hospital unit, and patient characteristics. Evidence that antibiogram-based CDSS improve empiric therapy is still limited (35), and traditional CDSS tools using static antibiogram data may fail to reflect real-time or location-specific resistance trends. Newer AI-driven models generate dynamic antibiograms that integrate patient factors such as transplant status, prior colonization, and recent antibiotic use to tailor empiric therapy.

Ridgway et al. evaluated the weighted incidence syndromic combination antibiogram, which uses patient-specific variables to guide empiric therapy (36). Although not yet clinically validated, such tools could identify resistance trends, flag concerning phenotypes, stratify resistance by patient subgroup, and prompt stewardship review. Although advanced tools hold great promise, many hospitals, especially smaller or resource-limited centers, lack the infrastructure needed to implement them. Until these platforms are widely available, conventional antibiograms will remain a foundational resource.

Antibiograms are essential instruments for safeguarding patient care and preserving antimicrobial effectiveness. Without accurate, timely, and well-analyzed antibiogram data, hospitals lose their ability to detect emerging resistance, target interventions, and measure the impact of stewardship and infection control programs. Rather than being replaced, they are more likely to be adapted and incorporated into modernized, data-driven approaches to antimicrobial decision support.

**Zoe Freeman Weiss**
